# 5-Hy­droxy-6-[(*E*)-2-phenyl­ethen­yl]-5,6-dihydro-2*H*-pyran-2-one isolated from *Goniothalamus ridleyi*

**DOI:** 10.1107/S1600536812028334

**Published:** 2012-06-30

**Authors:** Samsiah Jusoh, Laily B. Din, Zuriati Zakaria, Hamid Khaledi

**Affiliations:** a School of Chemical Sciences and Food Technology, Faculty of Science and Technology, National University of Malaysia, 43600 UKM Bangi, Selangor, Malaysia; b Department of Chemistry, University of Malaya, 50603 Kuala Lumpur, Malaysia

## Abstract

In the title compound, C_13_H_12_O_3_, the pyran ring adopts a half-chair conformation with a C atom deviating from the least-squares plane of the remaining ring atoms by 0.606 (2) Å. This plane and that of the benzene ring make a dihedral angle of 44.18 (6)°. In the crystal, mol­ecules are linked through O—H⋯O hydrogen bonds into infinite chains along the *b* axis, and these chains are cross-linked by C—H⋯O hydrogen bonded into sheets lying parallel to the *bc* plane. The layers are further connected *via* C—H⋯π inter­actions to form a three-dimensional supra­molecular structure.

## Related literature

For spectroscopic characterization of the 5β-hy­droxy­goniothalamin, see: Goh *et al.* (1995 ▶). For the crystal structures of some similar compounds, see: Fun *et al.* (1995 ▶); Tuchinda *et al.* (2006 ▶).
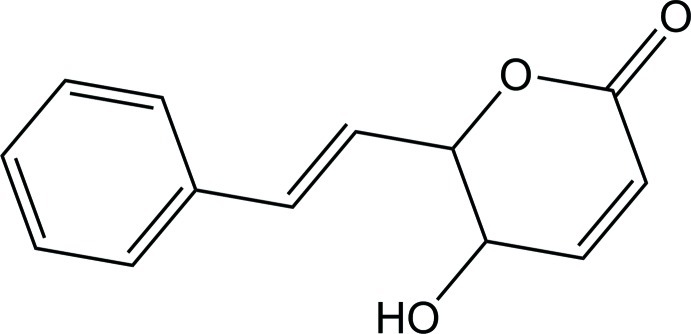


## Experimental

### 

#### Crystal data

C_13_H_12_O_3_
*M* *_r_* = 216.23
Monoclinic, 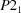

*a* = 6.5442 (8) Å
*b* = 11.0267 (14) Å
*c* = 8.0991 (10) Å
β = 111.402 (2)°
*V* = 544.14 (12) Å^3^
*Z* = 2
Mo *K*α radiation
μ = 0.09 mm^−1^
*T* = 100 K
0.30 × 0.18 × 0.06 mm

#### Data collection

Bruker APEXII CCD diffractometer
Absorption correction: multi-scan (*SADABS*; Sheldrick, 1996 ▶) *T*_min_ = 0.973, *T*_max_ = 0.994
2559 measured reflections
1250 independent reflections
1220 reflections with *I* > 2σ(*I*)
*R*_int_ = 0.012

#### Refinement

*R*[*F* ^2^ > 2σ(*F* ^2^)] = 0.027
*wR*(*F* ^2^) = 0.069
*S* = 1.08
1250 reflections
148 parameters
1 restraint
H atoms treated by a mixture of independent and constrained refinement
Δρ_max_ = 0.18 e Å^−3^
Δρ_min_ = −0.18 e Å^−3^

### 

Data collection: *APEX2* (Bruker, 2007 ▶); cell refinement: *APEX2*; data reduction: *SAINT* (Bruker, 2007 ▶); program(s) used to solve structure: *SHELXS97* (Sheldrick, 2008 ▶); program(s) used to refine structure: *SHELXL97* (Sheldrick, 2008 ▶); molecular graphics: *X-SEED* (Barbour, 2001 ▶); software used to prepare material for publication: *SHELXL97* and *publCIF* (Westrip, 2010 ▶).

## Supplementary Material

Crystal structure: contains datablock(s) I, global. DOI: 10.1107/S1600536812028334/pv2562sup1.cif

Structure factors: contains datablock(s) I. DOI: 10.1107/S1600536812028334/pv2562Isup2.hkl

Supplementary material file. DOI: 10.1107/S1600536812028334/pv2562Isup3.cml

Additional supplementary materials:  crystallographic information; 3D view; checkCIF report

## Figures and Tables

**Table 1:** Hydrogen-bond geometry (Å, °) *Cg* is the centroid of the C1–C6 ring.

*D*—H⋯*A*	*D*—H	H⋯*A*	*D*⋯*A*	*D*—H⋯*A*
O1—H1*A*⋯O2^i^	0.87 (3)	1.95 (3)	2.8026 (19)	170 (2)
C12—H12⋯O1^ii^	0.95	2.53	3.427 (2)	157
C9—H9⋯*Cg* ^ii^	1.00	2.97	3.747 (2)	135
C10—H10⋯*Cg* ^iii^	1.00	2.80	3.6561 (18)	144

Symmetry codes: (i) 
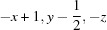
; (ii) 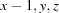; (iii) 
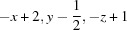
.

**Fig. 1.**
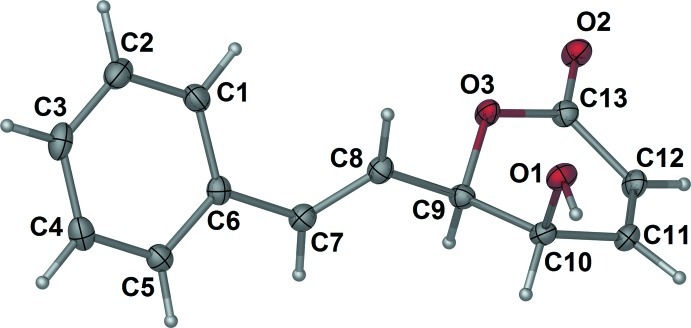
Molecular structure of the title compound with displacement ellipsoids drawn at 50% probability level. Hydrogen atoms are drawn as spheres of arbitrary radius.

**Fig. 2.**
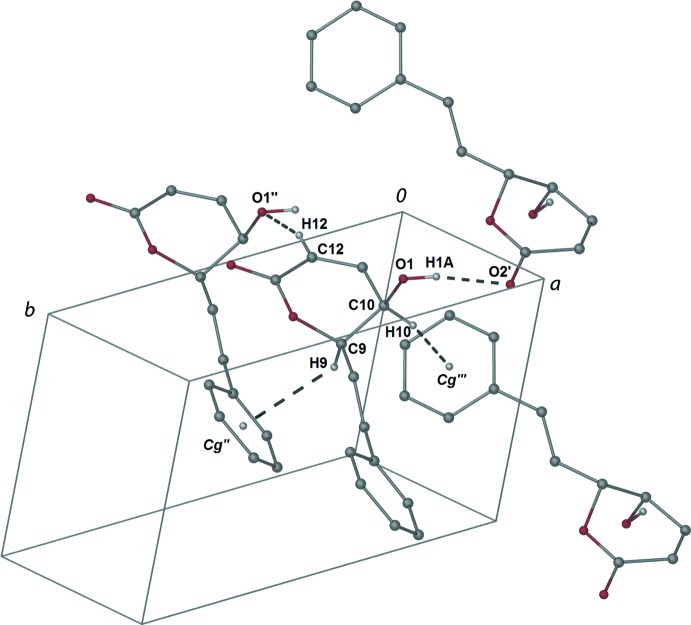
A view of the O—H···O, C—H···O and C—H···π interactions in the structure. Hydrogen atoms, except those involved in hydrogen bonding, are ommited. Symmetry codes: ' = -x + 1, y - 1/2; '' = x - 1, y, z; ''' = -x + 2, y - 1/2, -z + 1.

## supplementary crystallographic information

### Comment

The title compound was isolated from the roots of *Goniothalamus ridleyi* and found to be the same styrylpyrone isolated from the stem bark of *Goniothalamus dolichocarpus* (Goh *et al.*, 1995). In agreement with the structures of similar molecules (Fun *et al.*, 1995; Tuchinda *et al.*, 2006), the pyran ring in the title molecule adopts a half-chair conformation with C9 displaced by 0.606 (2) Å from the plane of the remaining ring atoms (C10/C11/C12/C13/O3). This plane and the benzene ring make a dihedral angle of 44.18 (6)°. The crystal packing comprises three dimensional network formed by O—H···O, C—H···O and C—H···π interactions (Table 1, Fig. 2).

### Experimental

Samples of the roots of *G. ridleyi* were collected from Post Brooke, Gua Musang, Kelantan, Malaysia. The roots were dried in an oven (323 K), ground and extracted using cool extraction. The extraction using three types of solvents *i. e*., hexane, chloroform and methanol gave three crude extracts. The chloroform crude extract (9.57 g) was separated using vacuum liquid chromatography (VLC). A mixture solvent of ethyl acetate and methanol as eluent solvent gave 12 fractions. TLC profiles showed fractions 1–3 were identical. Therefore, these fractions has been selected for further separation using column chromatography (CC) with eluent solvents hexane and ethyl acetate; 178 vials were collected and vials 157–165 have been selected for preparative TLC (PTLC) using hexane:ethyl acetate (9:11). GRAB 6 (0.0617 g) with *R*_f_ 0.46 in solvent system hexane: ethyl acetate (5:5) was crystallized from a mixture of ethyl acetate and n-hexane (1:1) at room temperature.

### Refinement

The C-bound hydrogen atoms were located in the calculated positions and refined in a riding mode with C—H distances of 0.95 (C*_sp2_*) and 1.000 (C*_sp3_*) Å. The O-bound H atom was found in a difference Fourier map and refined freely. For all hydrogen atoms, *U*_iso_ were set to 1.2*U*_eq_(carrier atom). In the absence of significant anomalous scattering effects Friedel pairs were merged.

### Crystal data

**Table:**

C_13_H_12_O_3_	*F*(000) = 228
*M**_r_* = 216.23	*D*_x_ = 1.320 Mg m^−^^3^
Monoclinic, *P*2_1_	Mo *K*α radiation, λ = 0.71073 Å
Hall symbol: P 2yb	Cell parameters from 1643 reflections
*a* = 6.5442 (8) Å	θ = 2.7–29.6°
*b* = 11.0267 (14) Å	µ = 0.09 mm^−^^1^
*c* = 8.0991 (10) Å	*T* = 100 K
β = 111.402 (2)°	Plate, colorless
*V* = 544.14 (12) Å^3^	0.30 × 0.18 × 0.06 mm
*Z* = 2	

### Data collection

**Table:**

Bruker APEXII CCD diffractometer	1250 independent reflections
Radiation source: fine-focus sealed tube	1220 reflections with *I* > 2σ(*I*)
Graphite monochromator	*R*_int_ = 0.012
φ and ω scans	θ_max_ = 27.0°, θ_min_ = 2.7°
Absorption correction: multi-scan (*SADABS*; Sheldrick, 1996)	*h* = −8→8
*T*_min_ = 0.973, *T*_max_ = 0.994	*k* = −12→14
2559 measured reflections	*l* = −10→10

### Refinement

**Table:**

Refinement on *F*^2^	Secondary atom site location: difference Fourier map
Least-squares matrix: full	Hydrogen site location: inferred from neighbouring sites
*R*[*F*^2^ > 2σ(*F*^2^)] = 0.027	H atoms treated by a mixture of independent and constrained refinement
*wR*(*F*^2^) = 0.069	*w* = 1/[σ^2^(*F*_o_^2^) + (0.0382*P*)^2^ + 0.0929*P*] where *P* = (*F*_o_^2^ + 2*F*_c_^2^)/3
*S* = 1.08	(Δ/σ)_max_ < 0.001
1250 reflections	Δρ_max_ = 0.18 e Å^−^^3^
148 parameters	Δρ_min_ = −0.18 e Å^−^^3^
1 restraint	Absolute structure: 749 Friedel pairs were merged
Primary atom site location: structure-invariant direct methods	

### Special details

**Table:**

Geometry. All e.s.d.'s (except the e.s.d. in the dihedral angle between two l.s. planes) are estimated using the full covariance matrix. The cell e.s.d.'s are taken into account individually in the estimation of e.s.d.'s in distances, angles and torsion angles; correlations between e.s.d.'s in cell parameters are only used when they are defined by crystal symmetry. An approximate (isotropic) treatment of cell e.s.d.'s is used for estimating e.s.d.'s involving l.s. planes.
Refinement. Refinement of *F*^2^ against ALL reflections. The weighted *R*-factor *wR* and goodness of fit *S* are based on *F*^2^, conventional *R*-factors *R* are based on *F*, with *F* set to zero for negative *F*^2^. The threshold expression of *F*^2^ > σ(*F*^2^) is used only for calculating *R*-factors(gt) *etc*. and is not relevant to the choice of reflections for refinement. *R*-factors based on *F*^2^ are statistically about twice as large as those based on *F*, and *R*- factors based on ALL data will be even larger.

### Fractional atomic coordinates and isotropic or equivalent isotropic displacement parameters (Å^2^)

**Table:**

	*x*	*y*	*z*	*U*_iso_*/*U*_eq_	
O1	0.6604 (2)	0.26455 (12)	−0.01641 (17)	0.0203 (3)	
H1A	0.720 (4)	0.194 (2)	−0.012 (3)	0.024*	
O2	0.12430 (19)	0.53996 (12)	−0.04165 (17)	0.0225 (3)	
O3	0.46365 (18)	0.48046 (11)	0.10232 (16)	0.0185 (3)	
C1	1.2436 (3)	0.59025 (18)	0.3976 (2)	0.0217 (4)	
H1	1.1293	0.6247	0.2994	0.026*	
C2	1.4539 (3)	0.63794 (18)	0.4495 (2)	0.0248 (4)	
H2	1.4827	0.7041	0.3862	0.030*	
C3	1.6224 (3)	0.58916 (18)	0.5938 (3)	0.0237 (4)	
H3	1.7666	0.6215	0.6292	0.028*	
C4	1.5782 (3)	0.49275 (18)	0.6858 (2)	0.0221 (4)	
H4	1.6928	0.4591	0.7846	0.027*	
C5	1.3677 (3)	0.44522 (17)	0.6346 (2)	0.0187 (3)	
H5	1.3388	0.3801	0.6997	0.022*	
C6	1.1977 (3)	0.49255 (16)	0.4877 (2)	0.0173 (3)	
C7	0.9754 (3)	0.43928 (17)	0.4324 (2)	0.0191 (3)	
H7	0.9309	0.4056	0.5219	0.023*	
C8	0.8338 (3)	0.43534 (17)	0.2661 (2)	0.0186 (3)	
H8	0.8795	0.4654	0.1752	0.022*	
C9	0.6060 (3)	0.38607 (15)	0.2147 (2)	0.0172 (3)	
H9	0.5705	0.3771	0.3242	0.021*	
C10	0.5697 (2)	0.26443 (16)	0.1191 (2)	0.0176 (3)	
H10	0.6423	0.1994	0.2070	0.021*	
C11	0.3265 (3)	0.23880 (16)	0.0389 (2)	0.0200 (4)	
H11	0.2771	0.1573	0.0154	0.024*	
C12	0.1802 (3)	0.32814 (17)	0.0004 (2)	0.0203 (4)	
H12	0.0282	0.3092	−0.0406	0.024*	
C13	0.2497 (3)	0.45594 (16)	0.0204 (2)	0.0179 (3)	

### Atomic displacement parameters (Å^2^)

**Table:**

	*U*^11^	*U*^22^	*U*^33^	*U*^12^	*U*^13^	*U*^23^
O1	0.0233 (6)	0.0155 (6)	0.0254 (6)	0.0005 (5)	0.0130 (5)	−0.0002 (5)
O2	0.0178 (6)	0.0187 (6)	0.0302 (7)	0.0013 (5)	0.0079 (5)	0.0025 (5)
O3	0.0136 (5)	0.0159 (6)	0.0237 (6)	−0.0004 (4)	0.0040 (4)	0.0009 (5)
C1	0.0229 (8)	0.0214 (9)	0.0180 (8)	0.0008 (7)	0.0042 (6)	0.0000 (7)
C2	0.0290 (9)	0.0222 (9)	0.0257 (9)	−0.0061 (8)	0.0130 (8)	−0.0031 (8)
C3	0.0175 (7)	0.0268 (10)	0.0282 (9)	−0.0057 (7)	0.0099 (7)	−0.0106 (8)
C4	0.0189 (8)	0.0226 (9)	0.0217 (8)	0.0044 (7)	0.0035 (6)	−0.0045 (7)
C5	0.0197 (8)	0.0184 (8)	0.0180 (8)	0.0023 (7)	0.0068 (6)	−0.0011 (7)
C6	0.0160 (7)	0.0180 (8)	0.0180 (7)	0.0004 (7)	0.0062 (6)	−0.0035 (7)
C7	0.0182 (8)	0.0184 (8)	0.0217 (8)	0.0001 (7)	0.0084 (7)	−0.0003 (7)
C8	0.0162 (7)	0.0177 (8)	0.0224 (8)	−0.0008 (7)	0.0078 (6)	−0.0008 (7)
C9	0.0161 (8)	0.0175 (8)	0.0179 (8)	0.0010 (6)	0.0059 (6)	0.0021 (6)
C10	0.0171 (7)	0.0154 (8)	0.0208 (8)	−0.0001 (6)	0.0075 (6)	0.0022 (7)
C11	0.0205 (8)	0.0163 (8)	0.0230 (8)	−0.0046 (7)	0.0077 (7)	−0.0006 (7)
C12	0.0128 (7)	0.0220 (9)	0.0241 (8)	−0.0039 (7)	0.0044 (7)	0.0003 (7)
C13	0.0153 (7)	0.0195 (9)	0.0204 (8)	−0.0005 (7)	0.0081 (6)	0.0005 (7)

### Geometric parameters (Å, º)

**Table:**

O1—C10	1.426 (2)	C5—H5	0.9500
O1—H1A	0.87 (3)	C6—C7	1.479 (2)
O2—C13	1.218 (2)	C7—C8	1.328 (2)
O3—C13	1.3399 (19)	C7—H7	0.9500
O3—C9	1.470 (2)	C8—C9	1.496 (2)
C1—C2	1.387 (2)	C8—H8	0.9500
C1—C6	1.394 (3)	C9—C10	1.523 (2)
C1—H1	0.9500	C9—H9	1.0000
C2—C3	1.390 (3)	C10—C11	1.510 (2)
C2—H2	0.9500	C10—H10	1.0000
C3—C4	1.388 (3)	C11—C12	1.329 (2)
C3—H3	0.9500	C11—H11	0.9500
C4—C5	1.388 (2)	C12—C13	1.471 (2)
C4—H4	0.9500	C12—H12	0.9500
C5—C6	1.400 (2)		
			
C10—O1—H1A	106.0 (15)	C7—C8—H8	118.3
C13—O3—C9	118.37 (13)	C9—C8—H8	118.3
C2—C1—C6	120.93 (16)	O3—C9—C8	104.91 (13)
C2—C1—H1	119.5	O3—C9—C10	111.27 (13)
C6—C1—H1	119.5	C8—C9—C10	114.59 (14)
C1—C2—C3	120.21 (18)	O3—C9—H9	108.6
C1—C2—H2	119.9	C8—C9—H9	108.6
C3—C2—H2	119.9	C10—C9—H9	108.6
C4—C3—C2	119.41 (16)	O1—C10—C11	109.78 (13)
C4—C3—H3	120.3	O1—C10—C9	111.02 (14)
C2—C3—H3	120.3	C11—C10—C9	109.15 (14)
C3—C4—C5	120.46 (16)	O1—C10—H10	109.0
C3—C4—H4	119.8	C11—C10—H10	109.0
C5—C4—H4	119.8	C9—C10—H10	109.0
C4—C5—C6	120.50 (16)	C12—C11—C10	121.20 (16)
C4—C5—H5	119.8	C12—C11—H11	119.4
C6—C5—H5	119.8	C10—C11—H11	119.4
C1—C6—C5	118.47 (15)	C11—C12—C13	121.12 (14)
C1—C6—C7	121.66 (15)	C11—C12—H12	119.4
C5—C6—C7	119.87 (15)	C13—C12—H12	119.4
C8—C7—C6	124.42 (16)	O2—C13—O3	118.41 (16)
C8—C7—H7	117.8	O2—C13—C12	123.25 (15)
C6—C7—H7	117.8	O3—C13—C12	118.22 (14)
C7—C8—C9	123.36 (16)		

### Hydrogen-bond geometry (Å, º)

Cg is the centroid of the C1-C6 ring.

**Table:**

*D*—H···*A*	*D*—H	H···*A*	*D*···*A*	*D*—H···*A*
O1—H1*A*···O2^i^	0.87 (3)	1.95 (3)	2.8026 (19)	170 (2)
C12—H12···O1^ii^	0.95	2.53	3.427 (2)	157
C9—H9···*Cg*^ii^	1.00	2.97	3.747 (2)	135
C10—H10···*Cg*^iii^	1.00	2.80	3.6561 (18)	144

Symmetry codes: (i) −*x*+1, *y*−1/2, −*z*; (ii) *x*−1, *y*, *z*; (iii) −*x*+2, *y*−1/2, −*z*+1.
